# One Anastomosis Gastric Bypass (OAGB) with a 150-cm Biliopancreatic Limb (BPL) Versus a 200-cm BPL, a Systematic Review and Meta-analysis

**DOI:** 10.1007/s11695-023-06556-9

**Published:** 2023-04-06

**Authors:** Mohamed AbdAlla Salman, Ahmed Salman, Mohamed Moustafa Assal, Mohammed Elsherbiney, Mohamed Tourky, Ahmed Elewa, Adel Mohamed Khalaf, Mohamed A. Gadallah, Mahmoud Gebril, Sadaf Khalid, Hossam Shaaban, Aboalgasim Alamin Mohammed, Mohamed Hosny Abdo Osman, Haitham Hassan

**Affiliations:** 1 KasrAlainy School of Medicine, Cairo, Egypt; 2 University Hospitals Dorset NHS Foundation Trust, Poole, UK; 3 United Lincolnshire NHS Trust, Lincoln, UK; 4 Great Western Hospitals, Swindon, UK; 5 National Hepatology and Tropical Medicine Research Institute, Cairo, Egypt; 6 Al_Azhar university, Assuit branch, Assuit, Egypt; 7 Glangwill General Hospital, Carmarthen, Wales, UK; 8 Royal Free London Hospital, NHS, London, UK; 9 North Cumbria Integrated Care NHS Foundation Trust, Cumberland Infirmary Hospital, Carlisle, UK

**Keywords:** Obesity, One anastomosis gastric bypass, Biliopancreatic limb length, 150 cm, 200 cm

## Abstract

This is a systematic review and meta-analysis that assessed the impact of performing OAGB with a 150-cm BPL versus a 200-cm BPL concerning weight loss, comorbidities remission, and adverse nutritional effects. The analysis included studies that compared patients who underwent OAGB with a 150-cm BPL and 200-cm BPL. Eight studies were eligible for this review after searching in the EMBASE, PubMed central database, and Google scholar. The pooled analysis revealed favoring the 200-cm BPL limb length for weight loss, with a highly significant difference in the TWL% (*p*=0.009). Both groups showed comparable comorbidities remission. Significantly higher ferritin and folate deficiency rates were found in the 200-cm BPL group. Considering a 200-cm BPL when performing OAGB delivers a better weight loss outcome than a 150-cm BPL, which is at the expense of a more severe nutritional deficiency. No significant differences were found regarding the comorbidities’ remission.

## Introduction

Obesity has been a pandemic with a continuously rising prevalence all over the world [1]. The only certain solution for severe obesity and its associated comorbidities in patients who are unable to lose weight through lifestyle modification and non-surgical means is bariatric surgery [2].

One anastomosis gastric bypass (OAGB) is one of the most widely accepted bariatric surgery procedures owing to its simplicity and proposed efficacy and safety [3, 4]. Currently, it comes just after sleeve gastrectomy and Roux-en-Y gastric bypass surgery (RYGB) [5]. OAGB comprises the creation of a long narrow gastric tube that undergoes side-to-side or end-to-side gastrojejunostomy. This anastomosis is formed at approximately150- to 200-cm distal to the Treitz ligament [6].

It has been claimed that OAGB is advantaged by its simplicity and the easiness of revision and reversion in addition to the fewer potential sites for internal hernia or leakage [7].

Up until now, no optimal biliopancreatic limb (BPL) length has been standardized in the OAGB operation. An improperly long BPL can elevate the risk of postoperative excessive loss of weight and developing nutritional deficiencies [8]. There is considerable variation in the BPL length customized by bariatric surgeons during OAGB [9]. Different BPL lengths have been studied to achieve a satisfactory weight loss with the least risk as far as possible [10]. Although a constant BPL length of 200 cm was the most commonly used [11], it has been presumed that a BPL length of 150 cm is the ideal [12].

This systematic review and meta-analysis aimed to assess the impact of performing OAGB with a 150-cm BPL versus a 200-cm BPL in terms of weight loss, comorbidities remission, and adverse nutritional effects.

## Methods

### Study Design

This is a systematic review and meta-analysis that was conducted following the Preferred Reporting Items for Systematic Reviews and Meta-Analyses (PRISMA) statement [13]. This analysis included studies that compared patients who underwent OAGB with a BPL length of 150 cm and those who had a BPL length of 200 cm. The search was performed using electronic resources: the EMBASE, PubMed central database, and Google scholar.

### Selection Strategy and Criteria

The search was conducted with restriction of results to original articles published until the time of analysis. The search was performed using the medical subject headings (MeSH) terms: “one anastomosis gastric bypass” OR “one-anastomosis gastric bypass” OR “single anastomosis gastric bypass” OR “single-anastomosis gastric bypass” OR “mini gastric bypass” OR “mini-gastric bypass” AND “biliopancreatic limb length” OR “bilio-pancreatic limb length” OR “BPL length” OR “BP limb length” AND “effect” OR “impact” OR “difference” OR “outcome” OR “risks” OR “benefits” OR “advantages” AND “150 cm” AND “200 cm.”

The search was performed by two independent reviewers (the first and second authors). Then, articles were matched and screened to ensure eligibility.

- Inclusion Criteria

Original articles available in English, those are addressing the effect of different BPL lengths in patients undergoing OAGB.

- Exclusion Criteria

Reviews, commentaries, and general discussion papers that do not present original data were excluded. Studies that do not contain a comparison between BPL lengths of 150 cm and 200 cm were also excluded.

### Data Extraction, Collection, and Analysis

The included articles were carefully read and the relevant data were extracted, registered, and analyzed. The included studies were evaluated for the encountered bias using the “Cochrane Collaboration”s tool for assessing the risk of bias.”

### Summary Measures

The primary outcome was the difference between 200-cm BPL and 150-cm BPL when performing OAGB, in the impact on weight loss outcome. The secondary outcome was the differences in comorbidities remission and nutritional deficiency rates.

### Statistical Analysis

The retrieved data were presented, analyzed, and tabulated. The meta-analysis and assessment of bias were performed using the Review Manager Software (RevMan version 5.4, the Cochrane Collaboration, London, UK). Numerical data were compared with the mean differences in effects between both groups, which were pooled into weighted mean differences (WMDs). Categorical data were presented as odds ratios and 95% confidence intervals (CIs). The percentage of variance in the meta-analysis was indicated by the *I*^2^ statistic to assess the heterogeneity between studies. Fixed- and random-effect models were used accordingly.

## Results

**Fig. 1:**
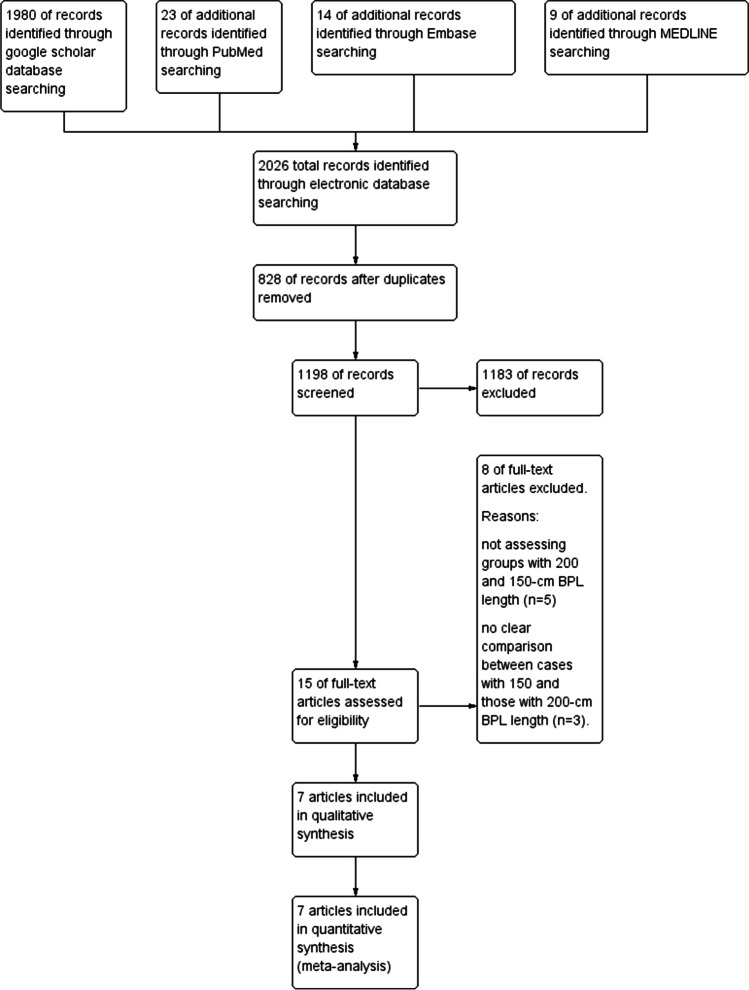
The included studies flow chart

The initial research on the electronic resources yielded 2026 records. After adjusting the duplications, the search provided 1199 results. The articles’ titles and abstracts were checked and 1183 articles were excluded. Reading the full texts of the remaining 16 articles resulted in the inclusion of 7 articles. One study was available as a poster abstract only [14]. However, considering the published data, the poster was included in the review. Thus, finally, 8 studies were eligible for this systematic review [9, 14–20]. Figure 1 demonstrates the included studies flow chart. One of the included articles [18] was an additional report of a previously published study [16]. Therefore, we omitted the repeated data and kept those concerning different outcomes.

The included articles were all recent. They were published from 2019 to 2022. The populations were patients scheduled for OAGB. They underwent mean follow-up periods ranging from 10 to 44.87 months. However, in most of the studies, the median follow-up period was 24 months [9, 15–17, 19]. The sample size of the included studies ranged from 155 [19] to 784 [20]. All the included studies were retrospective analyses for prospectively registered hospitals databases.

Five out of the sex included studies categorized their cohort into two groups: 200-cm BPL group and 150-cm BPL group. In the 3 other studies, there was an additional group: a 180-cm BPL group [9, 17] and a 250-cm BPL group [19].

The total population number of the studies included in the current review was 2599: 1100 (42.3%) underwent OAGB with a 150-cm BPL and 1336 (51.4%) underwent OAGB with a 200-cm BPL. The remaining 111 patients (4.3%) underwent OAGB with variable BPL lengths and were not included in the analysis.

The characteristics of the included studies and the differences between the 200-cm BPL and 150-cm BPL groups in the weight loss, comorbidities remission, and nutritional state are shown in Tables 1 and 2.

**Table 1:** The included studies and patients characteristics

Study	Boyle and Mahawar [15]	Omar et al. [16]	Pizza et al. [17]	Jedamzic et al. [19]	Slagter et al. [9]	Sam et al. [18]	Samuel et al. [14]	Bertrand et al. [20]
Year	2020	2021	2020	2020	2021	2022	2019	
Type of study	Retrospective analysis of the prospective hospital database							
Follow-up (months)	24	24	24	24	24	36	12	32.4–44.87
150-cm group (*n*)	118	171	60	11	172	171	178	392
200-cm group (*n*)	225	234	60	93	72	234	310	392
Both groups (*n*)	343	405	180	155	244	405	488	784
Females: *n* (%)	232 (67.6)	275 (67.9)	117 (65%)	111 (71.6)	199 (81.6)	275 (67.9)	327 (67)	598 (76.3)
Mean age	46.3±12.8	46 ± 10.98	35.2 ± 9	45 ± 4.5	48 ± 11	46 ± 10.98	46.5±7.25	44±11.3
Baseline weight	137.6	139±29.96	119.9±28.9	128.5 (92–196)	124±17	139±29.96	NA	121±16, 120±16.6
Baseline BMI	48.39	49±8.14	44.93±7.56	45.1 (33.1–71.1)	44±4	49±8.14	44±2.75	43±3.6, 42.6±3.6
200-cm group EWL%	75± 20.1	NA	61.2 ± 12.1	82.2 ± 24.8	75 (59–81)	76.46±20.1	68 (53–83)	76.8 ± 21.6
150-EWL% mean	74± 22	NA	60.7 ± 16.1	63.2 ±17.0	83 (65–99)	75.02+21.35	67 (53–80)	75.5 ± 24.02
200-TWL% mean	36.1 ± 9.2	NA	41.8 ± 8.9	34.5 ± 9.4	34 (28–38)	36.15±9.19	NA	NA
150-TWL% mean	34 ± 9.8	NA	40.7 ± 9.4	33.1 ± 5.2	29 (23–36)	34.12+9.49	NA	NA
Diabetes mellitus resolution: *n* (%)								
200-cm group	29 (46)	NA	5 (50)	NA	13 (87)	NA	NA	45 (48.4)
150-cm group	12 (46.2)	NA	5 (45.5)	NA	21 (68)	NA	NA	38 (50.7)
Diabetes mellitus improvement: *n* (%)								
200-cm group	24 (38)	NA	3 (30)	NA	2 (13)	NA	NA	NA
150-cm group	13 (50)	NA	2 (18.2)	NA	10 (32)	NA	NA	NA
Hypertension resolution: *n* (%)								
200-cm group	28 (33.7)	NA	17 (53.1)	NA	10 (48)	NA	NA	33 (21.9)
150-cm group	21 (42.9)	NA	18 (52.9)	NA	28 (49)	NA	NA	31 (22.3)
Hypertension improvement: *n* (%)								
200-cm group	24 (28.9)	NA	NA	NA	11 (52)	NA	NA	NA
150-cm group	11 (22.4)	NA	NA	NA	25 (42)	NA	NA	NA
Reoperation: *n* (%)								
200-cm group	2 (0.89)	NA	1 (1.67)	NA	NA	NA	NA	31 (7.9)
150-cm group	0 (0)	NA	2 (3.33)	NA	NA	NA	NA	16 (4.1)

**Table 2:** Comparison between both groups in the nutrients deficiency rates

Authors	Boyle and Mahawar [15]	Omar et al. [16]	Pizza et al. [17]	Jedamzic et al. [19]	Slagter et al. [9]	Sam et al. [18]	Samuel et al. [14]	Bertrand et al. [20]
Low serum protein: *n* (%)								
200-cm group	NA	NA	5 (59.6)	23 (38.3)	NA	NA	NA	NA
150-cm group	NA	NA	3 (5.7)	2 (28.6)	NA	NA	NA	NA
Low serum albumin: *n* (%)								
200-cm group	2 (0.92)	3 (2.53)	5 (3.8)	2 (3.3)	NA	3 (2.53)	NA	25 (6.4)
150-cm group	2 (1.8)	4 (2.29)	2 (1.9)	2 (2.9)	NA	4 (2.29)	NA	9 (2.3)
Low serum ferritin: *n* (%)								
200-cm group	NA	18 (14.75)	4 (21.1)	19 (33.9)	NA	NA	NA	65 (16.6)
150-cm group	NA	14 (11.97)	7 (13.4)	1 (14.3)	NA	NA	NA	38 (9.7)
Low serum folate: *n* (%)								
200-cm group	NA	18 (13.24)	NA	2(3.6)	NA	NA	NA	24 (6.1)
150-cm group	NA	11 (9.6)	NA	0 (0)	NA	NA	NA	12 (3.1)
Low serum vitamin B12: *n* (%)								
200-cm group	NA	1 (0.71)	4 (7.6)	1 (1.7)	NA	NA	NA	27 (6.9)
150-cm group	NA	0 (0)	3 (5.7)	0 (0)	NA	NA	NA	26 (6.6)
High serum parathyroid hormone: *n* (%)								
200-cm group	NA	64 (46.38)	NA	15 (26.3)	NA	NA	NA	NA
150-cm group	NA	45 (40.91)	NA	3 (42.9)	NA	NA	NA	NA
Low serum vitamin D: *n* (%)								
200-cm group	NA	6 (4.41)	7 (13.4)	45 (76.3)	NA	NA	NA	NA
150-cm group	NA	4 (3.57)	6 (11.5)	6 (85.7)	NA	NA	NA	NA

Regarding studies outcomes, these were mainly the effect of different BPL lengths on weight loss and comorbidities resolution [9, 14, 15, 17, 20]. Other outcomes were the effect on haematinics [16], micronutrients [16, 17, 19] and liver function tests [18].

In all studies, the basic preoperative weight, comorbidities, and nutritional status were comparable in the study groups.

**Fig. 2:**
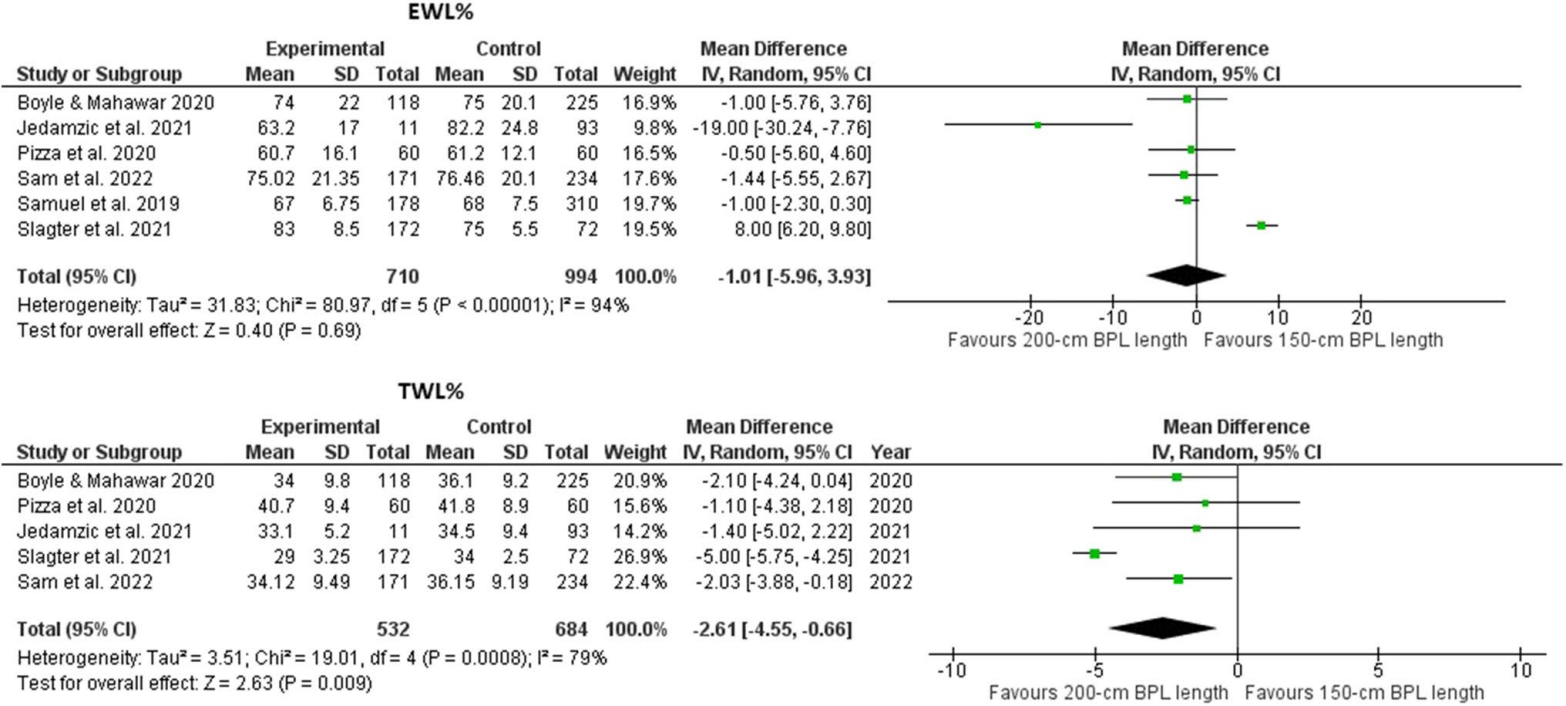
Foster plots for the EWL% and TWL% in the included studies

The pooled analysis revealed favoring the 200-cm BPL limb length for weight loss, with a statistically non-significant difference in the EWL% (*p*=0.67) and a highly significant difference in the TWL% (*p*=0.009) (Fig. 2).

**Fig. 3:**
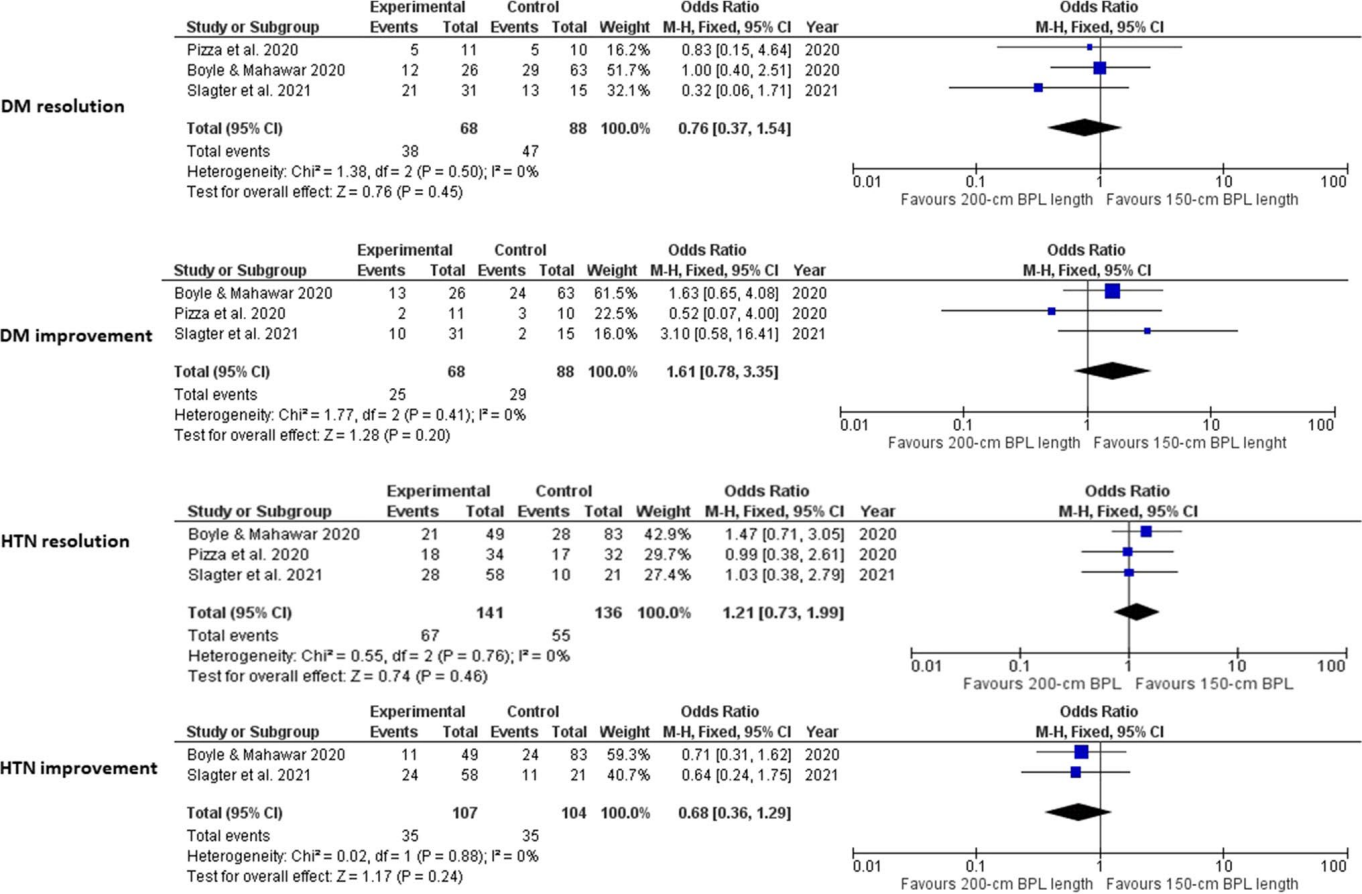
Foster plots for diabetes mellitus and hypertension complete resolution/improvement in the included studies

No statistically significant differences were found between the two groups in the percentages of diabetes mellitus resolution (*p*=0.78) or improvement (*p*=0.2). Likewise, no statistically significant differences were noted in hypertension resolution (*p*=0.54) or improvement (*p*=0.24) percentages (Fig. 3).

**Fig. 4:**
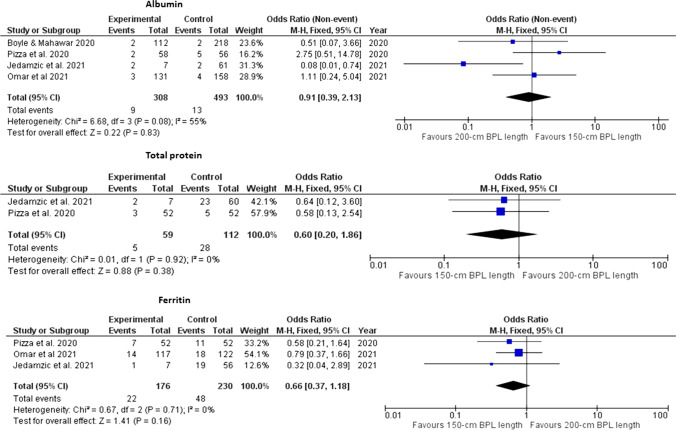
Foster plot for hypoalbuminemia, low protein levels, and low ferritin levels in the included studies

**Fig. 5:**
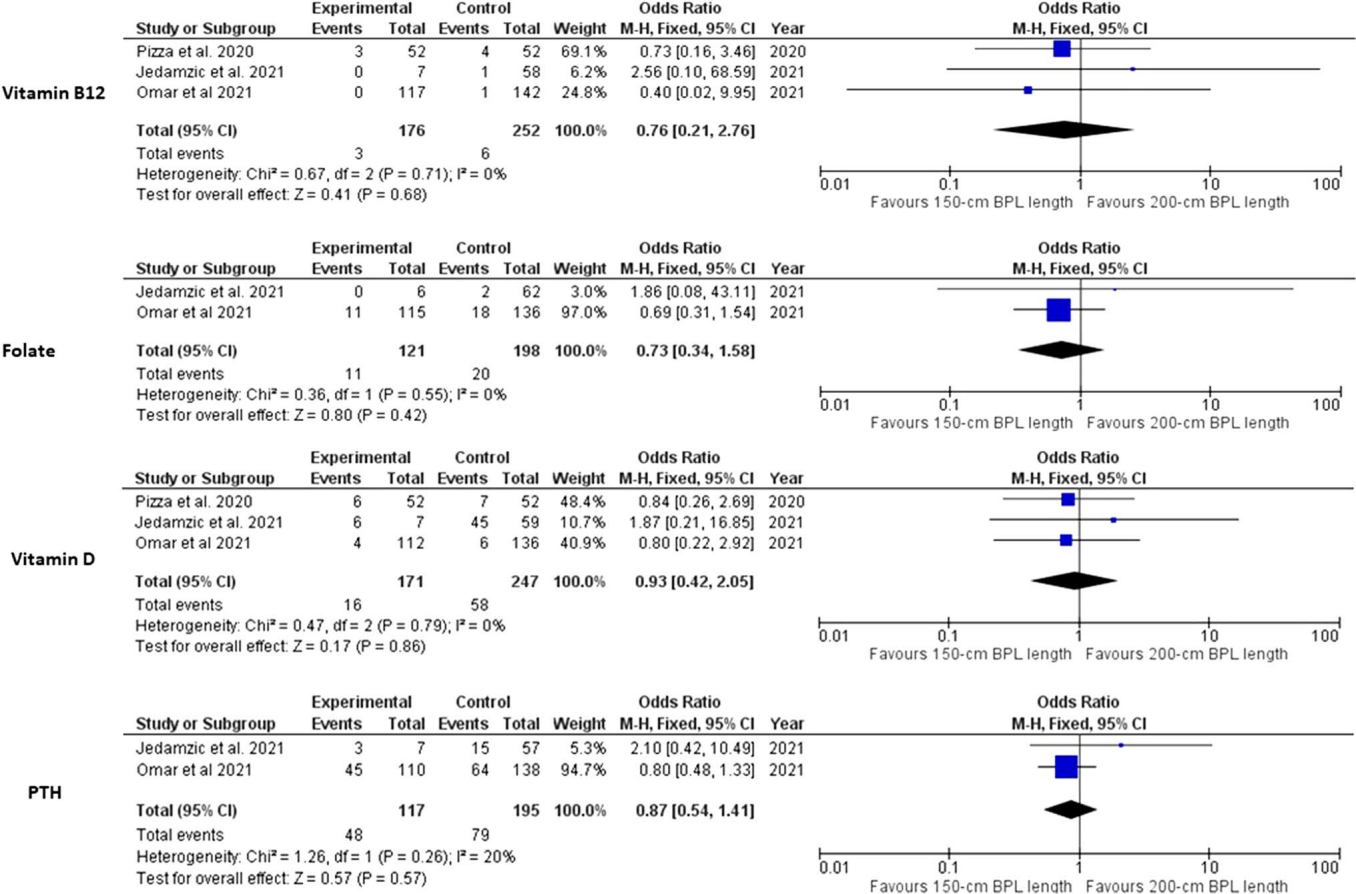
Foster plot for low vitamin B12, folate, and vitamin D levels and high postoperative reoperation rate in the included studies

There was statistically significant difference between the two groups in the postoperative ferritin (*p*=0.002) and folate (*p*=0.04) deficiency. Otherwise, no statistically significant differences was found between the two groups in the abnormal levels of any of the other studied nutrients. These findings were obtained from pooled analyses of 2 to 5 studies. The studied nutrients were albumin [9, 16, 17, 19, 20], total protein [17, 19], ferritin [16, 17, 19, 20] (Fig. 4), vitamin B12 [16, 17, 19, 20], folate [16, 19, 20], vitamin D [16, 17, 19] and parathyroid hormone [16, 19] (Fig. 5).

**Fig. 6:**
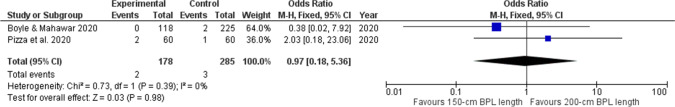
Foster plot for 30-days postoperative reoperation rate in the included studies

The reoperation rates were described in 4 studies [9, 15, 17, 20] (Fig. 6), their meta-analysis revealed non-significant difference between the two groups (*p*=0.13).

**Fig. 7:**
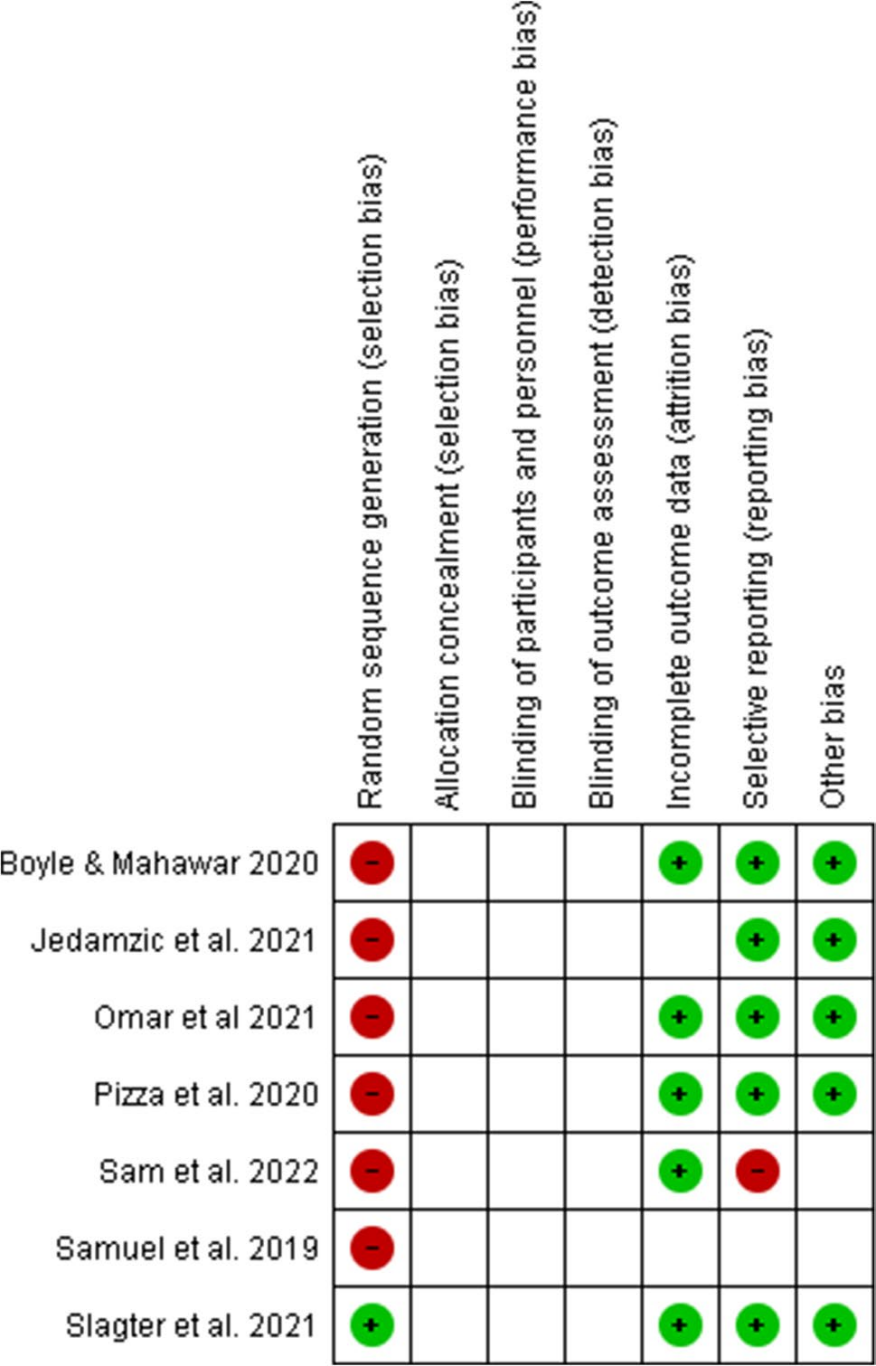
Review authors’ judgments about each risk of bias item for each included study

The critical assessment graph and summary of the risks of bias within each study, as thought by the authors, are shown in Fig. 7.

### Summary of the Included Studies

Boyle and Mahawar compared weight loss outcomes, comorbidities remission, hemoglobin and albumin levels, and morbidity/mortality of 118 patients undergoing OAGB with a 150-cm BPL with 225 patients with a 200-cm BPL. They found that both groups showed similar outcomes and concluded that a BPL of 150 cm is not inferior to that of 200 cm.

The study of Omar et al. [16] estimated the nutritional status of 234 patients with OAGB-200 cm and 171 with OAGB-150 cm. They found that micronutrients deficiency (vitamins A, B12, D, and E, folate, albumin, parathyroid hormone, and ferritin) occurred in both groups, with a higher incidence in the 200-cm group.

Pizza et al. [17] assessed the difference between both lengths in the weight loss outcome, comorbidities remission, nutritional status, and gastroesophageal reflux disease. They evaluated 180 patients distributed equally among 150-cm, 180-cm, and 200-cm groups. They did not note significant differences in any of the studied parameters apart from ferritin deficiency rates, which differed significantly between the 150-cm and 200-cm groups.

Sam et al. [18] studied the effect of both lengths on hepatic functions derangement**.** They explored the OAGB safety in terms of its effect on liver functions, with no significant difference between both limb lengths.

Slagter et al. [9] evaluated three BPL lengths groups (150-cm, 180-cm, and 200-cm) concerning differences in the weight loss outcome. Their published figures display higher TWL% in 200-cm BPL patients. Nevertheless, longer BPL did not deliver higher comorbidities remission rates.

Jedamzik et al. [19] evaluated the impact of BPL length in OAGB on protein and micronutrients deficiency. They assessed patients with 150-, 200-, and 250-cm BPL lengths. They reported that systemic levels of vitamins (A, B12, D, and E), folate, albumin, parathyroid hormone, calcium, iron, ferritin, and magnesium were comparable in patients with 150-cm and 200-cm BPL lengths. In patients with 250-cm BPL length, folate level was significantly lower when compared to patients with other limb lengths (150 and 200 cm).

In their published poster, Samuel et al. [14] compared weight loss in patients undergoing OAGB with a 150 cm to those with a 200-cm BPL. No significant differences in BMI, diabetes mellitus, hypertension, obstructive sleep apnea, or gastroesophageal reflux disease improvement were found.

Bertrand et al. [20], in their single-center retrospective study of 784 patients, compared patients who underwent OAGB with a 200-cm BPL to patients with OAGB with a 150-cm BPL in terms of weight loss and late morbidity. They used propensity score matching method to match patients in 1:1 ration based on age, sex, and BMI. They found no significant difference in the early morbidity. Regarding nutritional deficiencies, the 150-cm group showed a significantly lower percentage of hypoalbuminemia, low vitamin B9, and low ferritin. There was no significant difference in the EWL%.

## Discussion

For long-term treatment of obesity, procedures including malabsorption with restriction are likely superior to those restrictive only [21]. OAGB is one of the procedures combining malabsorption with restriction. Hence, it has gained acceptance for the treatment of obesity and its associated comorbidities. The International Federation for the Surgery of Obesity and Metabolic Disorders (IFSO) has recently acknowledged it as a mainstream bariatric surgery procedure [22]. Unfortunately, there is still no standardized technique to perform OAGB, with the length of BPL being the main debating item. Actually, there has been strong controversy considering different BPL lengths [23].

Despite using different BPL lengths by surgeons, a length of 200 cm has been the most commonly used [11, 24–26]. It was adopted by Rutledge [27] who first introduced the OAGB technique. Notwithstanding, several studies advocate for a 150-cm BPL to minimize the nutritional deficiencies with keeping an acceptable weight loss and comorbidities remission [28–32].

Only one meta-analysis could be reached in this context. Tasdighi et al. [33] performed a comparison between < 200-cm and ≥ 200-cm BPL lengths. However, in view that the latest IFSO Consensus Conference has recommended a BPL of 200 cm or less for OAGB to achieve a balance between effectiveness and safety, the current review was an attempt to evaluate which of the IFSO recommended lengths would be better.

The present work, to our knowledge, is the first meta-analysis comparing OAGB using a 200-cm BPL to a 150-cm BPL.

This review found that 200-cm BPL displayed better weight loss outcomes with a significant difference in the TWL%. There was comparable comorbidities remission. Nutritional deficiency rates were higher in patients with a 200-cm BPL.

The cause of the TWL% significant difference between the two groups is that all included studies showed higher TWL% in patients with 200-cm BPL [9, 15, 17–19]. Moreover, one of the included large-sized studies (*n*=244) [9] showed a high median difference (34 in 200-cm BPL group vs. 29 in 150-cm BPL group).

The mechanisms underlying superior weight loss outcomes with longer BPL are still indistinct. A longer BPL leads to bypassing larger area of the jejunum, with subsequent early nutrients malabsorption, leading to more loss of weight [34].

The included studies were unanimous concerning the comparable comorbidities remission effect of both limb lengths [9, 15, 17, 20]. This is explaining the overall non-significant difference found in this review. The overall hypertension remission (complete resolution or improvement) was encountered in 79.6% of patients with 150-cm BPL and 74.1% of patients with 200-cm length. This rate is in harmony with previous reports [7, 35, 36]. Bariatric surgery associated glycemic control has been extensively documented. The overall diabetes mellitus remission was found to occur in 92.6% of patients with 150-cm BPL compared to 86.4% in patients with 200-cm length. This rate aligns with Buchwald et al. [37] meta-analysis, who reported a ≥ 80% diabetes mellitus remission rate after OAGB. The 150-cm BPL-related higher remission rates may be attributed to that diabetes mellitus remission is prompted by several factors, such as age, baseline BMI and HBA1c levels, disease duration, and type of medications [33]. These factors are difficult to be adjusted for a reliable comparison.

The significantly higher ferritin and folate deficiency in the 200-cm BPL group denotes the higher malabsorption impact related to the longer BPL. The lack of significance in other nutrients’ deficiency in this analysis is likely due to the fact that all studies that assessed the nutritional state showed deficiency in both groups, either preoperatively, as a consequence of obesity-related nutritional disorders, or postoperatively, as operative sequels.

It is believed that OAGB owns a significant malabsorptive element since it somewhat acts as a biliopancreatic diversion, with a lack of the potentiality for digestion and absorption as food does not contact with the bypassed small bowel at all [38]. Interestingly, the randomized controlled trial introduced by Robert et al. [39] compared the outcomes of OAGB with a BPL of 200 cm versus standard Roux-en-Y gastric bypass (RYGB) with a BPL of 150 cm. They found that OAGB was related to weight loss and metabolic improvement comparable to that of RYGB, with higher incidences of malabsorption and nutritional adverse events. Similarly, Carbajo et al. [40] studied 1200 patients who were submitted to laparoscopic OAGB. The authors individualized the BPL length that ranged from about 200 cm up to 350 cm according the patient’s small bowel length and the BMI (tailoring technique). Nevertheless, only 1.1% of the patients suffered malnutrition. This was explained by the strict postoperative regimen followed by the investigators. In their malnutrition cases, the condition was temporary and responded to a strict program of enteral supplementation and counseling.

The current analysis reveals that the 200-cm BPL lengths had a superior weight loss outcome, namely the percentage of total weight loss, yet with a more severe nutritional deficiency. The lower weight loss outcome attributed to a 150-cm BPL is clinically questionable, with a mean TWL% difference of 2.6% and a mean EWL% of 0.9%. However, our findings imply the proper selection of compliant patients and the implementation of a strict surveillance program if the patients will undergo OAGB with a 200-cm BPL.

Some shortcomings should be acknowledged in the current systematic review including the small number of included studies, which is justified by the shortage of studies addressing this issue, and the lack of standardization of the follow-up period, where we tried our best to fix a follow-up period of 24 months to report the data whenever possible. In addition, all the included studies were retrospective analyses for a prospectively obtained hospital database. Unfortunately, no randomized controlled trials were available. Finally, we were obliged to include a poster abstract since it included valuable data regarding the weight loss outcome of a relatively large population number. Nevertheless, our study has the strength of being the first meta-analysis addressing these two limb lengths for more dedicated specification and standardization of the procedure technique within the IFSO recommendation window.

## Conclusion

Considering a 200-cm BPL when performing OAGB delivers a better weight loss outcome than a 150-cm BPL, which is at the expense of a more severe nutritional deficiency. No significant differences were found regarding the comorbidities’ remission.
